# Postcoital Internal Carotid Artery Dissection Presenting as Isolated Painful Horner Syndrome: A Case Report

**DOI:** 10.1155/2013/403647

**Published:** 2013-02-28

**Authors:** Eren Gozke, Hilal Tastekin Toz, Pınar Kahraman Koytak, Funda Alparslan

**Affiliations:** Department of Neurology, FSM Teaching and Research Hospital, 34752 Istanbul, Turkey

## Abstract

Postcoital artery dissection is a rare condition. Here we report a 40-year-old male patient with painful Horner syndrome related to postcoital internal carotid artery (ICA) dissection. In neurologic examination of the patient, semiptosis, enophthalmus, and myosis were observed on the left side. There were no carotid bruits. On T1-weighted and fat-suppressed cranial MRI, hyperintensity consistent with intramural hematoma was observed within cervical and temporal petrous segments of left ICA. On cervical and cranial MRA, marked decrease in the calibration of C1 and C2 segments of the left ICA was remarkable. The patient was diagnosed as left ICA dissection and anticoagulant therapy was initiated. A prominent improvement was noted in clinical findings during two months of followup period.

## 1. Introduction

Carotid artery dissection is characterized by the formation of mural hematoma as a result of rupture of tunica intima with an incidence of 2.5–3/100.000. It can develop spontaneously or secondary to trauma. Although postcoital artery dissections are rarely reported in the literature, any incident of postcoital carotid artery dissection in patients presented with isolated Horner syndrome has not been encountered so far [1, 2]. A case with postcoital internal carotid artery (ICA) dissection in a patient referred with painful Horner syndrome is presented.

## 2. Case Report

**Figure 1:**
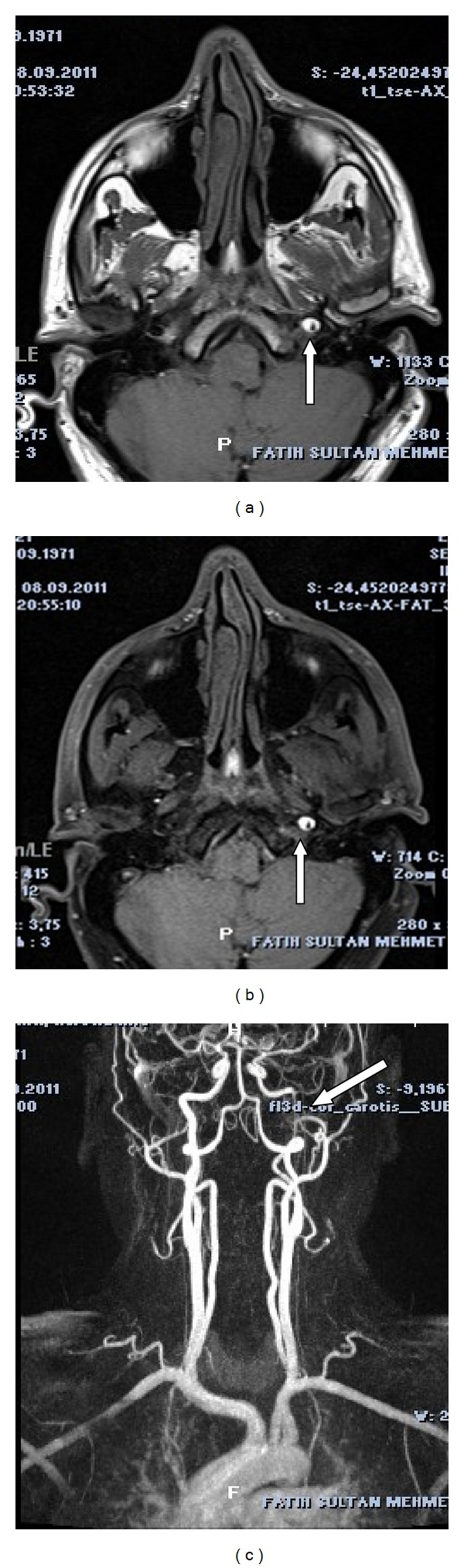
(a) Hyperintense area surrounding the left ICA on T1-weighted axial cranial MRI (white arrow). (b) Intramural hematoma on the left ICA on T1-weighted, fat-suppressed axial cranial MRI (white arrow). (c) Marked decrease in blood flow within cervical and temporal petrous segment of the left ICA on cervical MRA (white arrow).

 A 40-year-old male patient was referred to our outpatient clinic with complaints of rapid onset of pain localized on behind his left eye during intercourse and subsequent development of ptosis of his left eyelid. Physical examination did not reveal any evidence of abnormality. In neurologic examination, semiptosis, enophthalmus, and myosis were observed on the left side. There was no sweating abnormality on his face. Ophthalmologic evaluation revealed normal visual acuity and intact fundi, bilaterally. There were no carotid bruits. He was smoking a pack per day for 20 years, without any history of alcohol and any known substance and drug abuse. Cranial magnetic resonance imaging (MRI) and craniocervical magnetic resonance angiography (MRA) were performed on the patient with diagnosis of left Horner syndrome. On diffusion MRI, any acute ischemic lesion was not detected. On T1-weighted and fat-suppressed cranial MRI, hyperintensity consistent with intramural hematoma was observed within cervical and temporal petrous segments of left ICA (Figures 1(a) and 1(b)). On cervical and cranial MRA, marked decrease in the calibration of C1, and C2 segments of the left ICA was remarkable (Figure 1(c)). Any abnormal contrast enhancement in cerebral parenchyma was not observed after administration of contrast material. Any abnormality in routine laboratory test and markers of vasculitis was not detected. The patient was diagnosed as left ICA dissection, and anticoagulant therapy was initiated. First intravenous heparin was given, and then warfarin was started. A prominent improvement was noted in clinical findings during two months of followup period.

## 3. Discussion

Horner syndrome manifests itself with signs of ptosis, myosis, enophthalmus, and facial anhidrosis. It is characterized by interruption of oculosympathetic pathway at any point between hypothalamus and the eye. Denervations of pupillary dilator and Muller muscle result in myosis and ptosis, respectively. Ciliospinal reflex is lost. Facial flushing can be seen. Since sympathetic fibers responsible for sudomotor innervation diverge from oculosympathetic pathway before superior cervical ganglion, anhidrosis is not seen in postganglionic lesions [3]. Horner syndrome can be classified in three groups according to the location of the lesion. Central type can occur in lesions of hypothalamus, brain stem, and medulla spinalis which affect descendant sympathetic pathways. Most frequently seen types are lateral medullary syndrome (Wallenberg syndrome) and syringomyelia. Other concomitant neurologic findings might suggest this type. Preganglionic type results from root avulsions in medulla spinalis, brachial plexus lesions, traumas, pulmonary, and mediastinal tumors (Pancoast syndrome: pulmonary apical tumors, and concomitant shoulder-arm pain). Postganglionic type is related to internal carotid artery diseases, basis cranii lesions, cavernous sinus diseases, superior orbital fissure, or orbital apex diseases [4]. 

Although patients with spontaneous ICA dissections frequently present with clinical manifestations of stroke, only 20% of the patients seek medical care with local complaints of head and neck pain, pulsatile tinnitus, and symptoms of isolated Horner syndrome. In the literature, in 91% of the cases with Horner syndrome developed secondary to ICA dissection, ipsilateral pains on neck and also headache have been reported. More rarely lower cranial nerve palsies can be seen [5, 6]. In untreated cases, the risk of early onset of ischemic stroke within the first two weeks is increased. It is critically important to consider carotid dissection in the differential diagnosis of the patients presented with painful Horner syndrome in order to initiate therapy as soon as possible and also prevent potential onset of ischemic stroke. There is no clear data about preference of anticoagulant or antiplatelet treatments [7]. The choice of treatment should be made in individual basis. 

## 4. Conclusion

Painful Horner syndrome with carotid artery dissection is a medical emergency in that carotid artery dissection is a serious consideration among its etiologic factors. In patients with Horner syndrome, attentive evaluation of medical history, physical, and radiological examination results is crucial so as to administer optimal and timely treatment and also prevent further deterioration of the patient's clinical state.
